# miR-320a Regulates Placenta Endothelial Function After Fetal Cardiopulmonary Bypass via the ATG7-SIRT1/FOXO1 Pathway

**DOI:** 10.1007/s11596-025-00115-2

**Published:** 2025-10-08

**Authors:** Yun Teng, Miao Tian, Xiao-kang Luo, Qiu-ping Jiang, Hai-yun Yuan, Jian Zhuang, Ji-mei Chen, Cheng-bin Zhou

**Affiliations:** 1 Department of Cardiovascular Surgery, Guangdong Cardiovascular Institute, Guangdong Provincial People’s Hospital, Guangdong Academy of Medical Sciences, Southern Medical University, Guangzhou, 510080 China; 2 Guangdong Provincial Key Laboratory of South China Structural Heart Disease, Guangzhou, 510080 China

**Keywords:** miR-320a, ATG7, Fetal cardiopulmonary bypass, SIRT1, FOXO1, Endothelial cells, Placenta, Autophagy, Endothelial dysfunction

## Abstract

**Objective:**

Placental dysfunction induced by fetal cardiopulmonary bypass (CPB) imposes limitations on the clinical application of this procedure. The potential impact of microRNA-mediated autophagy in placental endothelial cells on overall placental function remains elusive, necessitating a comprehensive exploration of the underlying mechanisms involved.

**Methods:**

We established fetal sheep CPB models and employed immunohistochemistry to assess the placental expression of ATG7. Bioinformatic analysis, coupled with dual-luciferase reporter assays, was used to elucidate the intricate relationship between miR-320a and ATG7. Changes in ATG7 expression were further investigated through Western blotting and quantitative polymerase chain reaction (qPCR). Human umbilical vein endothelial cells (HUVECs) were cultured, and in vitro experiments were conducted to evaluate their regulatory effects on endothelial function. Immunoblotting was used to measure the expression levels of ATG7, endothelin-1 (ET-1), SIRT1, and FOXO1, whereas enzyme-linked immunosorbent assay (ELISA) was used to quantify nitric oxide (NO) production.

**Results:**

Sixty minutes after CPB, a substantial decrease in ATG7 expression in placental tissue was observed. The downregulation of ATG7 expression led to increased ET-1 production in HUVECs, concomitant with decreased NO production. miR-320a was identified as a specific regulator of ATG7 expression, with subsequent experiments demonstrating a significant reduction in placental ATG7 levels upon injection of the miR-320a agomir compared with the miR-320a antagomir during fetal sheep CPB. In HUVECs, miR-320a downregulated ATG7, resulting in increased ET-1 production and diminished NO production. Treatment with the miR-320a mimic/miR-320a inhibitor revealed that miR-320a inhibited the SIRT1/FOXO1 pathway in HUVECs by downregulating ATG7 expression, culminating in increased ET-1 production and reduced NO levels.

**Conclusion:**

The observed downregulation of placental ATG7 expression subsequent to fetal CPB is intricately associated with endothelial dysfunction. Furthermore, our findings underscore the specific regulatory role of miR-320a in modulating ATG7 expression within the placenta. At the cellular level, increasing the level of miR-320a has emerged as a potential strategy for modulating endothelial function through the inhibition of ATG7 and the SIRT1/FOXO1 pathway.

## Introduction

Congenital heart disease (CHD) is the most common birth defect in newborns, and complex CHD (e.g., single ventricle, pulmonary atresia, aortic atresia, tricuspid atresia, and transposition of the great arteries) is the leading cause of neonatal mortality [1]. The correction of early fetal heart structural malformations to avoid subsequent pathological changes in the major blood vessels of the heart is an important strategy for reducing the occurrence of complex CHD and improving the success rate of neonatal treatment. Early correction of the structural anomalies of the fetal heart has not been widely adopted. Only a small number of centers have carried out intrauterine interventions for some heart valve diseases, and such interventions have high surgical risks and low success rates. Surgical repair to restore valve function is often required after birth [2, 3]. Fetal cardiac surgery is considered the most effective method for treating fetal cardiac lesions. However, there is still a lack of safe and effective fetal cardiopulmonary bypass (CPB) technology in clinical practice due to unavoidable postoperative complications.

Fetal CPB is a nonphysiological perfusion pattern, and its most common complication is placental dysfunction, which is characterized by increased vascular resistance, decreased blood flow, and ultimately impaired fetal gas exchange, which can lead to postoperative miscarriage [4]. The relevant pathophysiological changes include prostaglandin release, endothelial dysfunction, leukocyte and complement activation, and the activation of other inflammatory pathways. Since umbilical blood vessels are not innervated, their dilation is regulated by the vasodilatory paracrine effect of the placental vascular endothelium [5]. Several substances regulate vascular tone in the umbilical cord and placental villi, including vasodilators (e.g., prostacyclin, nitric oxide [NO], and natriuretic peptides) and vasoconstrictors (e.g., thromboxane A2, endothelin-1 [ET-1], and angiotensin) [6–8].

Autophagy is an important mechanism by which vascular endothelial cells maintain normal physiological functions [9]. Studies have shown that the autophagy of vascular endothelial cells under shear stress can increase NO production and reduce ET-1 production, thus protecting vasodilation function [10]. Conversely, inhibiting autophagy leads to inflammation and the massive production of oxygen-free radicals, thus damaging the function of vascular endothelial cells [11]. Microribonucleic acids (miRNAs) are a class of endogenous noncoding small-molecule single-stranded RNAs approximately 18–26 nucleotides in length. MiRNAs usually regulate protein production by inhibiting the translation process at the posttranscriptional level or leading to the direct degradation of translation products [12] and may therefore be potential therapeutic targets. However, the role of autophagy in placental vascular endothelial cells after exposure to abnormal shear stress during fetal CPB is unclear. Targets need to be identified to affect the function of the endothelium.

In this study, we established fetal sheep CPB models and investigated changes in autophagy-related protein 7 (ATG7) expression in the placenta. The influence of ATG7 on endothelial function was detected. We identified miR-320a as a potential target that regulates ATG7 expression during fetal CPB. We also cultured umbilical vein endothelial cells to explore whether ATG7 affects the function of endothelial cells through the Sirtuin 1 (SIRT1)/forkhead box protein O1 (FOXO1) pathway. Our study provides a new basis for identifying intervention targets to reduce placental injury during fetal CPB.

## Materials and Methods

### Animals and Establishment of Fetal CPB Models

Pregnant small-tailed Han sheep with fetuses at 120–140 days of gestation (equivalent to approximately 240–280 days of gestation in humans [13]) were purchased from Guangdong Medical Laboratory Animal Center. The experimental protocol was approved by the Animal Ethics Committee of Guangdong Provincial People’s Hospital (No. KY-D-2021–271-02) and conducted according to the National Research Council (NRC) Guide for the Care and Use of Laboratory Animals (8th edition).

The steps to establish the CPB models were as follows: the pregnant sheep were fasted the day before surgery. Tracheal intubation was applied after preoperative sedation. Electrocardiogram monitoring was performed on the pregnant sheep, and the fetal sheep were located via ultrasound. The uterus was exposed via an abdominal midline incision. After the fetal sternum was palpated, the nearby uterine wall was divided to open the amniotic cavity. When one upper limb of the fetal sheep was visible, the axillary artery of the fetal sheep was exposed for puncture and catheterization, and the ambulatory blood pressure of the fetal sheep was monitored. The chest was opened via the thoracic midline incision. The pericardium was divided longitudinally to expose the main pulmonary artery and right atrium. After systemic heparinization (300 IU/kg, intravenous) with a target activated clotting time (ACT) > 400 s, the main pulmonary artery was cannulated, and a straight venous drainage tube was inserted into the right atrial appendage. With the placenta as the only oxygenator of the fetus, a regular fetal sheep CPB model was established. The CPB arterial-venous shunts were subsequently connected. A centrifugal pump (Revolution5, Sorin Group Italia S.R.L., Italy) was used to construct a fetal sheep heart bypass model at room temperature. The physiological hypoxic state of the fetal sheep was simulated at the same time. The duration of the CPB was 60 min for each fetal sheep. In the control group, a median incision was made in the fetal sheep without cannulation or CPB. Each group contained three animals.

### Cell Culture

In this study, human umbilical vein endothelial cells (HUVECs) at passages 3–5 purchased from ATCC were cultured in complete medium consisting of 10% Dulbecco’s modified Eagle’s medium (DMEM, Gibco, USA, 1% fetal bovine serum (FPS, Gibco), penicillin (10,000 U/mL)–streptomycin (10,000 μg/mL) (PS, Gibco) at 37 °C in a 5% CO_2_ incubator. After the cell fusion reached 70%–80%, the cells were digested and passaged with phosphate buffer saline (PBS) and trypsin–EDTA (0.25%) (Gibco) and used for subsequent cell experiments.

### Dual-Luciferase Reporter Assays

Wild-type and mutant dual-luciferase reporter gene expression vectors for HUVECs transcription factor *ATG7* 3' untranslated region (UTR) fragments were constructed to verify the molecular mechanism underlying miR-320a target regulation. The Primer Bioinformatics software predicted that miR-320a would target the 3' UTR of the *ATG7* gene. The reference sequence of miR-320a was obtained from the bioinformatics database micRNABase (http://www.micRNAbase.org/). The *ATG7* 3' UTR sequences were checked via the National Center for Biotechnology Information. The biological target gene prediction software TargetScan (www.targetscan.org) was used to analyze hsa-miR-320a and the predicted binding sites in the *ATG7* 3' UTR and thus obtain the binding site. The gene fragments were obtained via polymerase chain reaction (PCR) amplification and then inserted downstream of the dual-luciferase genes in the psiCHECK2 vector. After double digestion, agarose gel electrophoresis, and sequencing, wild-type and mutant *ATG7* 3' UTR recombinant expression vectors were successfully constructed, which were then cotransfected into HUVECs with a miR-320a mimic (50 nM) or inhibitor (100 nM) to detect luciferase activity and ATG7 expression levels via the Dual-Luciferase Reporter Assay System (Promega, USA).

### Immunohistochemistry

Following ligation of the placental base, the placenta was harvested, and subsequent to the removal of blood with PBS, a surgical blade was employed within the placentome region to meticulously section the placenta into 3 mm × 3 mm blocks. Care was taken to ensure the inclusion of both the arcade region and the villus/crypt region of the placentome regions within the tissue. The dissected placental fragments were then immersed and fixed in 10% formaldehyde for 24 h. Subsequently, routine paraffin embedding and sectioning were performed. The paraffin sections were immunostained via immunohistochemical antibodies (catalog number: G1211; LandMBio, China). The brownish-yellow area of ATG7 expression was captured by ImageJ software, and the relative area was calculated.

### Quantitative Real-Time PCR (qRT-PCR)

After the cells were collected, total RNA was extracted via the TRIzol method, and its purity and integrity were tested. Total RNA was reverse-transcribed to complementary deoxyribonucleic acid (cDNA) according to the manufacturer’s protocol (EasyScript First-Strand cDNA Synthesis SuperMix, TransGen Biotech, China). qRT-PCR was performed on a qPCR machine (ABI PRISM® 7500 Sequence Detection System, Applied Biosystems, USA) with SYBR Green qPCR SuperMix (11733–046, Invitrogen, Thermo Scientific, USA). 18S rRNA (112 bp) was used as the internal reference gene fragment. The relative gene expression levels were calculated via the comparative Ct (ΔΔCt) method), and statistical analysis was performed to determine significant differences between groups. The primers used were the *ATG7*-F primer (CCTTGGGTTGCAATGTAGCT) and the *ATG7*-R primer (CTTACCACCCCCTAGGCAAT).

### Western Blotting

Tissues or cells were lysed in RIPA buffer (P0013, Beyotime, China) supplemented with PhosSTOP (KGP602, KeyGen Biotech, China) and a protease inhibitor cocktail (P8340, Sigma Aldrich, USA). Lysates were resolved by gel electrophoresis and transferred to PVDF membranes. Antibodies against ATG7 (ab52472, Abcam, UK), ET-1 (ab2786, Abcam), SIRT1 (ab110304, Abcam), and FOXO1 (Abcam, ab179450) proteins were used for immunoblotting.

### Enzyme-Linked Immunosorbent Assay (ELISA)

An enzyme-linked immunosorbent assay (ELISA) was employed to detect the production of NO (A013-2-1, Nanjing Jiancheng Biological Engineering Research Center, China) according to the manufacturer’s instructions. Briefly, after NO is treated with oxygen and water to form nitrate and nitrite, the two can generate light red azo compounds in the presence of nitrate chromogenic agents, and the concentration of NO can be indirectly measured via colorimetry. The intensity of the signal was measured via a microplate reader (Thermo Fisher Scientific, Germany). Statistical analysis was performed to determine significant differences between groups.

### Statistical Analysis

Statistical analysis was conducted via SPSS 25.0 software (SPSS Inc., USA). The data were derived from independent biological replicates and are presented as the mean ± standard error of the mean (SEM). For continuous variables, comparisons between two groups were performed with the Mann‒Whitney *U* test, and comparisons among groups were performed with one-way analysis of variance (ANOVA) followed by the least significant difference (LSD) test for pairwise comparisons. *P* < 0.05 was considered statistically significant.

## Results

### Endothelial Dysfunction Was Related to Decreased Placental *ATG7* Expression After CPB in Fetal Sheep

**Fig. 1:**
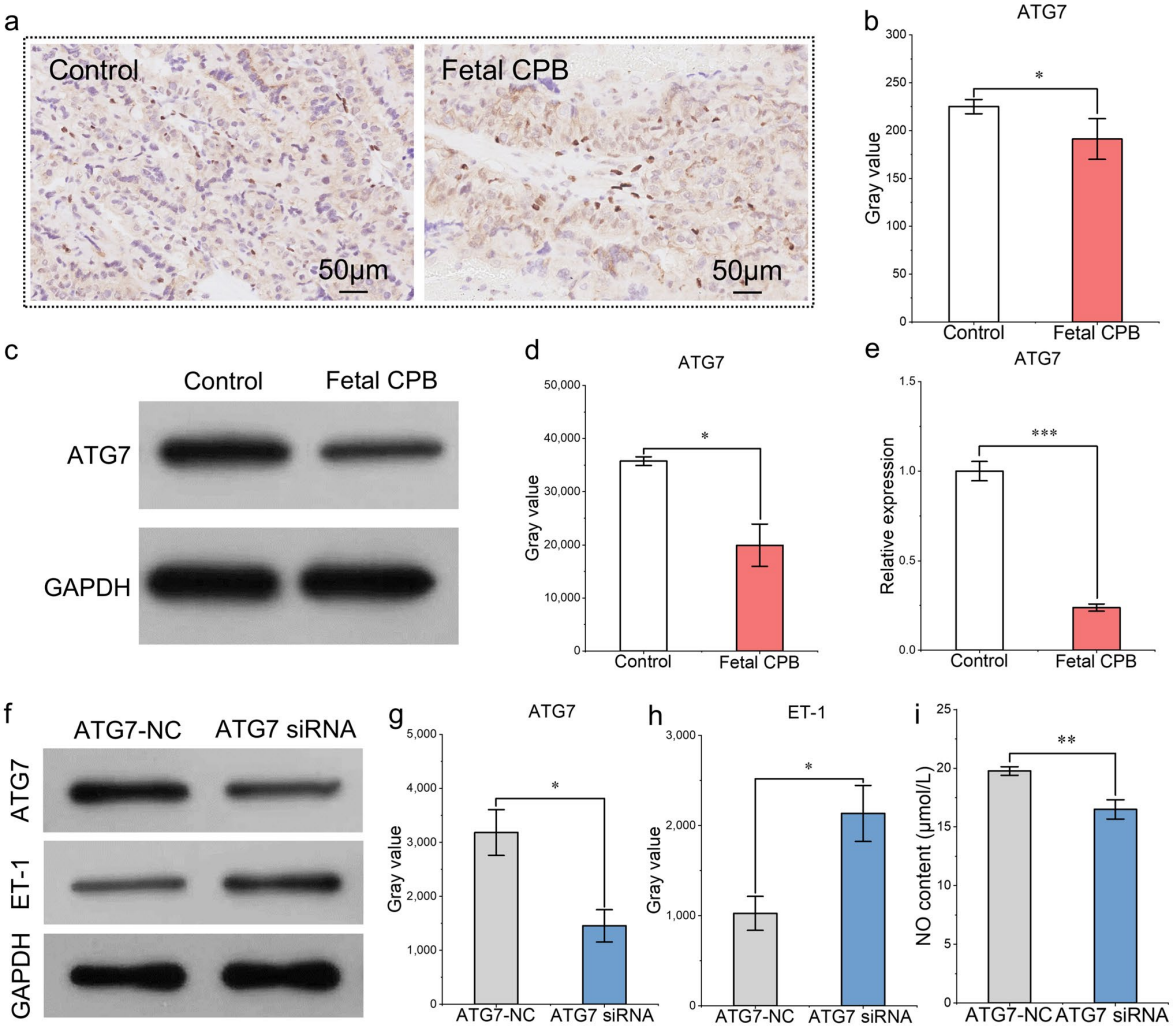
**a,** **b** Immunohistological detection and semiquantitative determination of autophagy-related protein 7 (ATG7) in paraffin sections from the control and simple cardiopulmonary bypass (CPB) groups. The ATG7 proteins were brown‒yellow after antigen‒antibody binding and diaminobenzidine (DAB) staining, and the nuclei were blue after hematoxylin staining. The brownish-yellow area of ATG7 expression was quantified via ImageJ software by calculating the background-subtracted integrated optical density (IOD) values. **c**, **d** Western blot results and grayscale analysis of ATG7 protein in the control group and the simple fetal CPB group. **e** qRT-PCR results of ATG7 in the control group and the fetal CPB group. **f**–**h** Western blot results and grayscale analysis of ATG7 and endothelin-1 (ET-1) proteins before and after the transduction of ATG7 siRNA into human umbilical vein endothelial cells (HUVECs). **i** Production of nitric oxide (NO) (μmol/L) before and after cotransfection of ATG7 siRNA into HUVECs (by ELISA). ^*^P < 0.05; ^**^P < 0.01; ^***^P < 0.001; n = 3

ATG7 is an important factor that regulates the autophagy of vascular endothelial cells [14]. The immunohistochemistry results for the paraffin sections of the placental tissues revealed that the protein level of ATG7 in the placental tissue significantly decreased after 60 min of simple fetal CPB compared with that in the control group (Fig. 1a, b). The results of Western blotting of placental tissue verified the immunohistochemical findings (Fig. 1c, d). The expression of the *ATG7* gene was also tested via qRT-PCR. The results revealed that the expression of ATG7 was reduced after fetal CPB, which was consistent with the Western blotting and immunohistochemical findings (Fig. 1e). Next, we explored the influence of decreased ATG7 on endothelial function in vitro. The HUVECs were cultured, and ATG7 siRNA (target sequence: GAACGAGTATCGGCTGGAT) was transduced to interfere with ATG7 expression. Western blotting was performed to detect the protein expression of ATG7, which revealed that the transfection efficiency was satisfactory (Fig. 1f, g). ET-1 was also tested by Western blotting, and the results revealed that ET-1 was increased in vascular endothelial cells after downregulating ATG7 expression (Fig. 1f, h). Moreover, NO production, as measured by ELISA, decreased with reduced ATG7 expression (Fig. 1i). Endothelial function was influenced by decreased ATG7.

### The Placental Expression of ATG7 Was Regulated by miR-320a

**Fig. 2:**
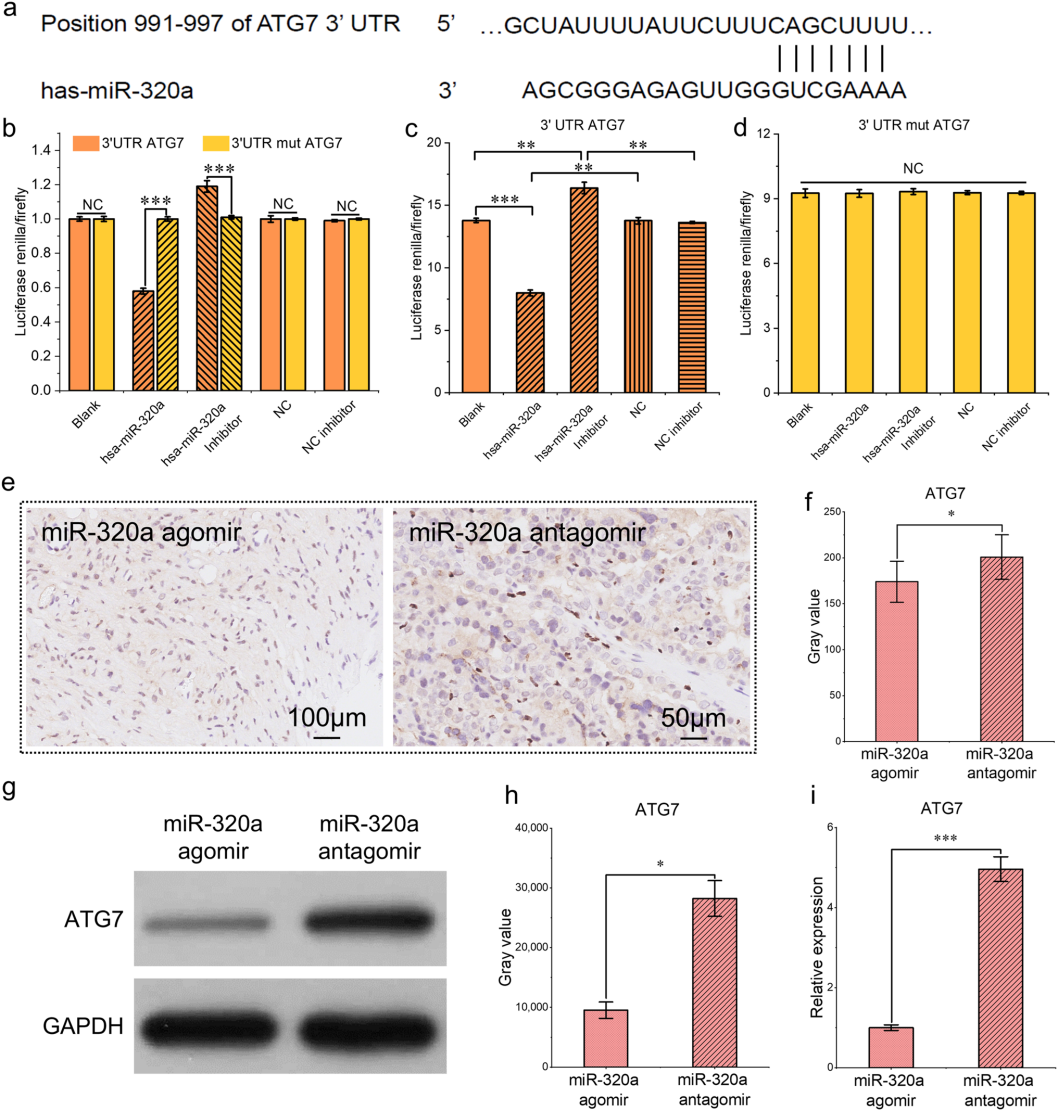
**a** Prediction of the binding site between ATG7 and miR-320a via TargetScan. **b**–**d** Evaluation of luciferase activity after the transfection of the ATG7 3' UTR and the ATG7 3' UTR mut. **e**, **f** Immunohistological detection and semiquantitative determination of ATG7 expression in paraffin Sections. Sixty min after the injection of the miR-320a agomir or antagomir during fetal CPB. The ATG7 proteins were brown‒yellow after antigen‒antibody binding and DAB staining, and the nuclei were blue after hematoxylin staining. **g**, **h** Detection of ATG7 by Western blotting and grayscale analysis after the injection of the miR-320a agomir or antagomir during fetal CPB. **i** qRT-PCR results of ATG7 expression 60 min after the injection of miR-320a agomir or antagomir. ^*^P < 0.05; ^**^P < 0.01; ^***^P < 0.001; n = 3

Several miRNAs have been shown to regulate the pathophysiological processes of placental vascular endothelial cells, among which miR-320a has been shown to induce apoptosis in endothelial cells in vitro while inhibiting the proliferation, migration, and invasion of these cells [15]. The bioinformatic analysis identified binding sites for hsa-miR-320a in the 3' UTR of the target gene *ATG7* (https://www.targetscan.org/, Fig. 2a). Dual-luciferase reporter assays revealed that the cotransfection of ATG7 and hsa-miR-320a reduced the luciferase activity by 42% relative to that of the control. The prediction of mutants revealed a relative value of 100% luciferase activity at the miR-320a binding sites (Fig. 2b). Has-miR-320a changed the fluorescence activity of ATG7 by binding to it. However, it did not affect the activity of mut-ATG7 by binding to the latter (Fig. 2c, d), indicating that miR-320a regulates ATG7 by binding to the 3' UTR in a targeted manner and that there is only one binding site.

To examine the effect of miR-320 on placental ATG7 expression during fetal CPB in sheep, we added miR-320a agomir or antagomir at the start of CPB via a single injection via the umbilical artery (200 nM, 50 mL). The immunohistochemistry (Fig. 2e, f) and Western blotting (Fig. 2 g–i) results for the paraffin sections revealed that the protein expression of ATG7 after 60 min of intervention with the miR-320a agomir was significantly lower than that after intervention with the miR-320a antagomir. Thus, placental ATG7 expression during CPB could be affected by miR-320a.

### miR-320a Regulates the Function of the Endothelium Through the SIRT1/FOXO1 Pathway

**Fig. 3:**
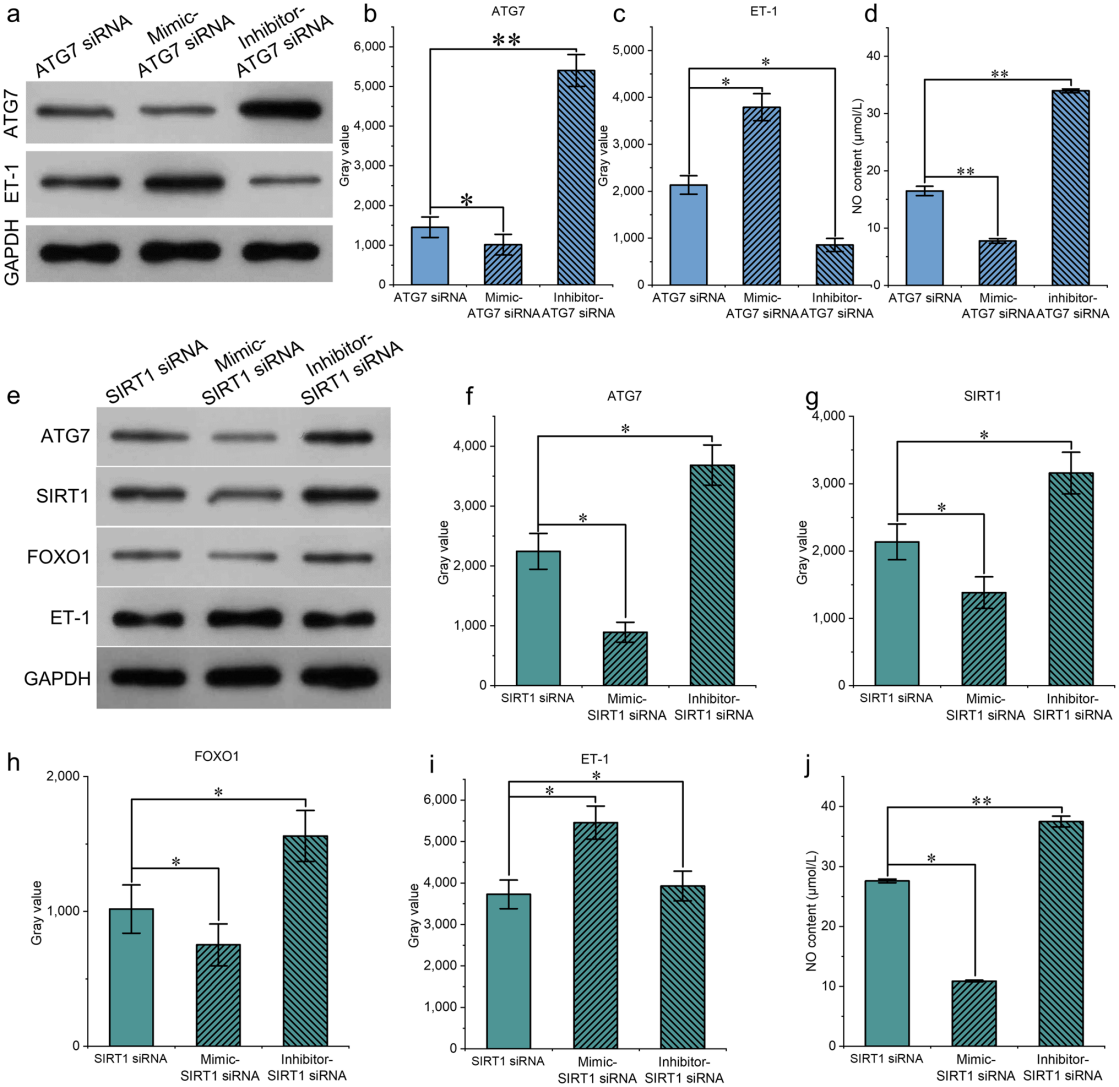
**a**–**c** Evaluation by Western blotting of ET-1 and ATG7 after the cotransfection of ATG7 siRNA with the miR-320a mimic or miR-320a inhibitor into HUVECs. **d** NO production (μmol/L) after cotransfection of ATG7 siRNA with the miR-320a mimic or miR-320a inhibitor into HUVECs was detected via ELISA. **e**–**i** Western blot results and grayscale analysis of ATG7, SIRT1, ET-1, and FOXO1 after cotransfection of SIRT1 siRNA with the miR-320a mimic or miR-320a inhibitor into HUVECs. **j** Production of NO (μmol/L) detected by ELISA after the cotransfection of SIRT1 siRNA with the miR-320a mimic or miR-320a inhibitor into HUVECs. ^*^P < 0.05; ^**^P < 0.01; ^***^P < 0.001; n = 3

Next, we explored the regulation of endothelial function by miR-320a in vitro. HUVECs were cultured. ATG7 siRNA was cotransduced with miR-320a-mimic/miR-320a-inhibitor. Western blotting revealed that ATG7 expression, which was reduced by ATG7 siRNA, was further decreased after transduction with the miR-320a mimic (Fig. 3a, b). ET-1 increased in endothelial cells with the downregulation of ATG7 expression (Fig. 3c), and NO production decreased with the reduction in ATG7 (Fig. 3d). However, after the addition of the miR-320a inhibitor, the elevated expression of ET-1 was significantly suppressed with the increasing levels of  ATG7 (Fig. 3c). NO production increased again after the addition of inhibitors of miR-320a (Fig. 3d), which suggests that miR-320a regulates the function of umbilical vein endothelial cells.

ATG7 has been shown to promote shear stress-induced SIRT1/FOXO1 pathway-mediated autophagy in vascular endothelial cells and to protect the function of vascular endothelial cells [16]. We explored whether miR-320a affects endothelial function through the same pathway. After the HUVECs were transfected with the SIRT1 siRNA, changes in the expression of ATG7, SIRT1, ET-1, and FOXO1 were detected via Western blotting, and ELISAs were used to detect NO production. After the interference of SIRT1, ET-1 expression significantly increased, FOXO1 expression decreased, and NO production decreased (Fig. 3d–j). The addition of the miR-320a mimic strengthened the effect of the SIRT1 siRNA. However, SIRT1 was upregulated after the addition of the miR-320a inhibitor (Fig. 3 g). NO production also increased with the upregulation of FOXO1 and ATG7 (Fig. 3j). Thus, miR-320a may regulate the function of endothelial cells through the SIRT1/FOXO1 pathway.

## Discussion

CHD is the most common birth defect, seriously affects the health and well-being of newborns, and increases the burden placed on families and society. The diagnosis and treatment of CHD have continued to improve, but complex CHD remains one of the leading causes of death in infants, especially in newborns [17]. Fetal echocardiography has greatly improved the prenatal diagnosis of complex CHD. For pregnant women who wish to keep their diseased baby, the intrauterine repair of fetal cardiac malformations to avoid secondary changes in the large blood vessels of the heart is a key strategy for increasing the success rate of treatment for complex CHD. Fetal cardiac interventions have been carried out in clinical settings in some hospitals, enabling a small proportion of fetuses with relevant indications to achieve biventricular development and thus avoiding the impact of the Fontan procedure after birth on the long-term quality of life and life expectancy of children with monoventricles [18]. Despite these advantages, interventional techniques still have some shortcomings and limitations, which limit their further application and efficacy. For some particular cases, fetal cardiac surgery might be a better intrauterine treatment for fetal heart abnormalities.

CPB is essential for fetal heart surgery. Before the introduction of CPB in human fetuses, its feasibility and effectiveness need to be explored in animal experiments. The number of fetuses in a pregnant sheep and the cardiovascular physiology of the fetal sheep are very close to those of humans; thus, most animal experiments on fetal CPB have been conducted in fetal sheep [13]. Early studies have shown that fetal CPB may affect placental function, making it difficult for fetal sheep to survive in utero. Recent studies have shown that a nonstop beating heart and physiologically hypoxic CPB impair umbilical cord placental circulation, causing placental dysfunction, which decreases the survival rate of fetal sheep in utero [19]. Changes in placental function are related to placental vascular dysfunction, and vascular endothelial cells are essential for the regulation of placental vascular function [20]. In the present study, we investigated the effect of CPB on placental vascular function in animal models and then explored the molecular mechanism underlying placental dysfunction.

Shear stress is the mechanical force generated by the sliding of blood across the endothelial surface. It is considered one of the factors that determines the quiescent state of endothelial cells [21]. Shear stress plays a key role in regulating biological functions, such as gene expression, proliferation, migration, morphogenesis, and permeability of endothelial cells, as well as thrombosis and inflammation. Endothelial cells transfer mechanical signals through a network composed of multiple messenger molecules and signaling proteins, but the exact mechanism by which this occurs remains unknown. Unidirectional wall shear stresses with relatively high magnitudes (e.g., shear stress generated by the laminar flow of arterial trees) are thought to have a vasculoprotective effect. Conversely, shear stresses associated with disturbances in blood flow (e.g., low shear stress and oscillating shear stress found in branched or curved areas) are triggers for endothelial cell dysfunction. In our previous studies, we reported that fetal placental function was impaired after the establishment of CPB, and the umbilical arterial pulsatility index also decreased with prolonged CPB [22]. We speculate that the abnormal shear stresses generated by the turn of blood flow during CPB result in dysfunction of placental vascular endothelial cells.

Different studies have shown that the level of autophagy in cells can be affected to varying degrees by blood shear forces. Guo et al*.* [10] examined the effects of stable laminar shear stress on the autophagy of vascular endothelial cells and the expression of endothelial NO synthase (eNOS) and ET-1 in endothelial cells via an ex vivo perfusion system. They reported that autophagy appeared to play a protective role in vascular endothelial cells and arteries cultured in an in vivo perfusion system by promoting the expression of eNOS and inhibiting the expression of ET-1. Yao et al*.* [23] found that laminar shear stress promoted autophagy in vascular endothelial cells by upregulating *Rab4*. On the basis of these previous studies, we speculated that abnormal shear stress may cause autophagy in placental vascular endothelial cells and thus lead to dysfunction, as confirmed in our current study. By monitoring changes in classical autophagy-related protein 7, we demonstrated the occurrence of autophagy in placental vascular endothelial cells. The observed increase in ET-1 and reduction in NO production indicate a shift toward vasoconstriction in the placental vasculature. This imbalance likely elevates placental vascular resistance, impairing umbilical blood flow and exacerbating gas exchange dysfunction during CPB. These hemodynamic alterations may have contributed to the placental insufficiency observed in our model.

Autophagy is a process in which eukaryotic cells use lysosomes to eliminate cytoplasmic proteins and damaged organelles under the regulation of autophagy-related genes. Autophagy includes basal autophagy under normal physiological conditions and induced autophagy under stress conditions. As a self-protection mechanism of cells, basal autophagy is beneficial for the growth and development of cells. It can protect cells from metabolic stress and oxidative damage, thus playing key roles in maintaining intracellular homeostasis and in regulating the synthesis, degradation, and recycling of cell products. However, excessive autophagy can lead to metabolic stress, degradation of cellular components, and even cell death. Studies have shown that autophagy can play an important role in various physiopathological processes, including cell homeostasis, aging, immunity, tumorigenesis, and neurodegenerative diseases [24]. As an indispensable autophagy effector enzyme, ATG7 regulates immunity, cell death, and protein secretion together with other ATG proteins and can independently regulate the cell cycle and apoptosis. Collier et al*.* [25] elucidated the roles of ATG7 in the pathogenesis of diseases in a variety of systems and organs, including the central nervous system, liver, pancreas, skeletal muscle, and circulatory system. ATG7 has also been shown to promote shear stress-induced SIRT1/FOXO1 pathway-mediated autophagy in vascular endothelial cells and protect their function. This study focused primarily on the autophagy-related functions of ATG7.

We found that ATG7 expression in the placenta decreased after CPB in fetal sheep, which might be regulated by miR-320a. Further experiments revealed that miR-320a mimics decreased NO release and increased ET-1 release in endothelial cells by downregulating ATG7, and these changes were reversed after miR-320a inhibitor treatment, which suggests that miR-320a affects changes in placental vascular function via ATG7. It is possible that miR-320a leads to increased ET-1 production and decreased NO production in endothelial cells by suppressing the SIRT1/FOXO1 pathway, thus affecting the functions of both vascular endothelial cells and the placenta. Although HUVECs are a standard endothelial model, differences in gene expression and shear responses exist between HUVECs and placental microvascular cells. Further studies with primary placental endothelia could enhance the clinical relevance of these findings.

## Conclusion

ATG7 expression in the placenta was downregulated after fetal CPB in sheep, which was related to endothelial dysfunction. The placental expression of ATG7 can be regulated by miR-320a in a targeted manner. At the cellular level, increasing miR-320a could regulate endothelial function by suppressing ATG7 and the SIRT1/FOXO1 pathway, resulting in increased ET-1 production and decreased NO production in endothelial cells.

## Data Availability

The data that support the findings of this study are available from the corresponding author on reasonable request.
